# Volume Targeted Ventilation and High Frequency Ventilation as the Primary Modes of Respiratory Support for ELBW Babies: What Does the Evidence Say?

**DOI:** 10.3389/fped.2020.00027

**Published:** 2020-02-07

**Authors:** Abhrajit Ganguly, Abhishek Makkar, Krishnamurthy Sekar

**Affiliations:** Section of Neonatal-Perinatal Medicine, University of Oklahoma Health Sciences Center, Oklahoma City, OK, United States

**Keywords:** ELBW, ventilation strategies, high frequency ventilation, volume targeted ventilation, extremely premature infants

## Abstract

Respiratory management of the extremely low birth weight (ELBW) newborn has evolved over time. Although non-invasive ventilation is being increasingly used for respiratory support in these ELBW infants, invasive ventilation still remains the primary mode in this population. Current ventilators are microprocessor driven and have revolutionized the respiratory support for these neonates synchronizing the baby's breath to ventilator breaths. High frequency ventilators with the delivery of tidal volumes less than the dead space have been introduced to minimize barotrauma and chronic lung disease. Despite these advances, the incidence of chronic lung disease has not decreased. There is still controversy regarding which mode is ideal as the primary mode of ventilation in ELBW infants. The most common modes seem to be pressure targeted conventional ventilation, volume targeted conventional ventilation and high frequency ventilation which includes high frequency oscillatory ventilation, high frequency jet ventilation and high frequency flow interrupter. In recent years, several randomized controlled trials and meta-analyses have compared volume vs. pressure targeted ventilation and high frequency ventilation. While volume targeted ventilation and high frequency ventilation does show promise, substantial practice variability among different centers persists. In this review, we weighed the evidence for each mode and evaluated which modes show promise as the primary support of ventilation in ELBW babies.

## Introduction

The rate of preterm birth (<37 weeks) in the USA has decreased between 2007 and 2014 by 8% (1). Extremely low birth weight (ELBW) babies occupy a large portion of the current population of preterm infants with gestational ages ranging from 22 to 28 weeks. With significant advances in the care of newborns, the high mortality among the ELBW population has been replaced by increased survival but with significant long-term morbidity. Several collaborative improvement projects and network studies have been undertaken between countries and centers with the evaluation of outcomes among the ELBW population. These major network studies have been published reporting these outcomes over time (2–5). While some reports show minimal improvement in certain outcome measures, others show worsening results. Given that major pulmonary morbidity and bronchopulmonary dysplasia (BPD) remain some of the most important outcome measures in this population, a lot of research has been done trying to optimize the respiratory management of ELBW babies. The principal components of respiratory management in ELBW babies include conventional ventilation (pressure or volume targeted), high frequency ventilation (HFV), non-invasive ventilation [nasal continuous positive airway pressure or nCPAP, nasal intermittent mandatory ventilation or NIMV, high flow or low flow cannula, non-invasive high frequency oscillatory ventilation or nHFOV and neurally adjusted ventilatory assistance or NAVA], use of surfactant and nitric oxide (3, 6, 7). While non-invasive ventilation could be an effective way of establishing functional residual capacity immediately after birth without the adverse effects of positive pressure ventilation, its use is limited to babies who are >25 weeks gestation (8). A majority of ELBW infants, however, require invasive ventilation. In 2012, The Neonatal Research Network found that 82% of the babies born before 29 weeks of gestation ended up needing invasive ventilation (3). These infants are also at the greatest risk of developing ventilation-induced lung injury (VILI) and BPD. While many recent advances have been made in neonatal ventilation strategies, the rate of BPD has either remained stable or increased depending on the region and centers (3, 9). An important reason for the increased prevalence of BPD is the increased survival especially at extreme gestational ages (22–24 weeks). The definition of BPD has evolved over the years with the most recent definitions including aspects, such as the gestational age, the total time on supplemental oxygen and the use of nasal CPAP or positive pressure ventilation (PPV) in addition to the dependence on oxygen at 36 weeks corrected gestational age (10). It is a well-established fact that lung injury secondary to invasive ventilation in addition to exposure to high oxygen plays an important role in the development of BPD. This is especially true in ELBW babies who tend to require invasive ventilation for a prolonged period. Ideally, it is beneficial for preterm babies to quickly transition from invasive to non-invasive modes of ventilation in order to minimize VILI. Bjorklund et al. found that even a few high tidal volume positive pressure breaths (35–40 ml/kg) shortly after birth was enough to initiate lung injury and minimize the effectiveness of later surfactant administration (11).

As our understanding of the different mechanisms of lung injury has improved along with the technological advancement of neonatal care, the mortality rate in the ELBW population has come down significantly. Early mortality in this age group at present is related more to sepsis, pulmonary hemorrhage, severe intracranial hemorrhage among others rather than acute respiratory failure (12). More babies in the ELBW population are surviving until discharge and as a result, decreasing later morbidity has become the focus for further research. Thus, we have seen large follow-up studies published in the last several years focusing on BPD and later pulmonary morbidities (13). Given the cascade of BPD is set into motion shortly after or even before the birth of the baby (14–16), proper respiratory management in the immediate post-natal period has become the focus of importance to reduce later morbidity. While the pathogenesis of BPD in ELBW babies may be multifactorial, ventilation is a component that is potentially modifiable (17). Therefore, it is of utmost importance to practice ventilation strategies that minimize lung injuries and in turn long-term morbidity and mortality. The use of volume targeted ventilation (VTV) and high-frequency ventilation (HFV) in extremely preterm infants have gained a lot of momentum in the last few decades because of their potential to minimize VILI (18, 19). This has resulted in some well-designed studies and meta-analysis being published which included ELBW babies (birth weight <1,000 g) (20–23). However, an international survey showed that the acceptance of these modes in the NICUs around the world is not consistent and carries significant practice variability (24). We have reviewed the existing literature including systematic reviews and meta-analysis for these popular modes of ventilation and explored whether a clear recommendation could be made regarding the primary modes of ventilatory management for ELBW infants.

## Conventional Ventilation In Elbw Infants

Conventional ventilation (CV) is a frequently used mode of invasive ventilation in ELBW babies (3). It is used in ELBW babies primarily for acute respiratory failure, which causes CO_2_ retention. Invasive mechanical ventilation can be administered in two different ways: pressure limited ventilation (PLV) and VTV. In PLV, a preset peak inspiratory pressure (PIP) is dialed above the positive end-expiratory pressure (PEEP) along with the rate and inspiratory time. The delivered tidal volume is variable depending on the change in lung compliance. As ventilation is based on constantly delivered tidal volume to the lung, PLV is not the ideal respiratory support for ELBW infants. VTV is based on the principle of targeting a specific tidal volume consistently during each breath. The tidal volume and respiratory rate set in the ventilator determine how efficiently the retained CO_2_ will be removed.

It is important to distinguish the concept of “volume targeted” ventilation from “volume control” ventilation in the context of neonatal respiratory management. These two terms have been used interchangeably by many authors over the years. Volume control ventilation is typically used in the adult and larger pediatric population. In this modality, a specific tidal volume (chosen by the user) is set and calculated at the ventilator machine itself and then administered through the circuit and finally to the patient (25). Thus, the pressure in the circuit rises throughout the breath and reaches the PIP just before expiration. In this mode, the pressure is a dependent variable to the set tidal volume. The rate of pressure rise also depends on the compliance of the lung and the resistance of the circuit. Additionally, because of the use of cuffed endotracheal tubes in this population, the amount of air-leak around the ETT is negligible. As a result, there is a good correlation between the calculated tidal volume at the ventilator and the administered tidal volume to the patient. This system would not work in the ELBW population because of the very low tidal volume required (~2–5 ml in babies <1,000 g) and the lost volume secondary to compression in the circuit. The situation is further complicated by the fact that air-leak around the ETT is variable between breaths thus making the correlation between the targeted tidal volume and the administered tidal volume very poor (26). This was a problem several years back when the ventilators were not sensitive enough to deliver small tidal volumes consistently with each breath (27). Earlier ventilators used to have flow sensors located in the main body of the ventilator (many ventilators still do) (26). Modern ventilators are equipped with a microprocessor that can detect small changes in tidal volumes as low as 0.5 ml (28, 29). The volume guarantee mode also makes these ventilators “self-weaning” in which inspiratory pressures are adjusted based on measured exhaled tidal volume, in response to changing lung compliance and respiratory effort (30, 31). This is especially helpful for preterm babies with respiratory distress syndrome who have received surfactant. The self-weaning features of these ventilators decrease the risk of volutrauma and unintended hyperventilation in these babies. Compared to PLV, VTV is associated with less variability in tidal volume and stable PaCO_2_ levels (31). Whereas, avoiding high tidal volume prevents volutrauma, avoiding lower than physiological tidal volume reduces the risk of atelectrauma and CO_2_ retention (32). Furthermore, avoiding both hypo and hypercarbia, prevent rapid changes in the cerebral blood flow thus decreasing the risk of intracranial bleeds, which is a major cause of morbidity and mortality in ELBW babies (33).

In VTV, the user-set tidal volume is measured at the airway opening (most commonly at the Y-piece between the ventilator circuit and the ETT) through a flow sensor, which makes it more accurate and sensitive in detecting changes in volume (28). The ventilator automatically adjusts the PIP on each administered breath to reach the preset tidal volume measured at the Y-piece. In essence, volume targeted ventilation is a “pressure control” mode of ventilation where tidal volume is a dependent variable to changes in pressure. Depending on the ventilator design or mode of VTV, the tidal volume may be measured at the flow sensor either during inspiration or during expiration or both. Expired tidal volume is generally considered more accurate as it is not affected by the air-leak around the ETT unless the leak is very high (>50%) (34). Nonetheless, volume control ventilation is still being used in many NICUs and Singh et al. showed that volume control ventilation can be used safely in extremely preterm infants when an additional flow sensor is used near the ETT which helps to adjust the volume according to the targeted tidal volume (35).

## Volume Targeted (Vtv) Vs. Pressure Limited Ventilation (Plv) In Elbw Infants

Numerous studies have been published comparing VTV and PLV in the neonatal population (36–39). However, there is very little data specifically on ELBW babies. A Cochrane meta-analysis was published in 2017 which included 16 parallel and 4 cross-over studies (20). Data from 977 infants were included in the analysis and the authors found that VTV offered multiple benefits when compared to PLV. VTV was found to significantly decrease rates of death and/or BPD, mean duration on ventilation, air leaks, hypocarbia, and severe intraventricular hemorrhage (IVH). Additionally, there was no significant difference in patent ductus arteriosus (PDA) and inspired oxygen concentration between the two groups. However, there were considerable differences between studies in regard to the diagnostic criteria for PDA and oxygen targeting strategies. The authors, however, report the quality of evidence for these outcome measures is moderate to low due to the use of different ventilators and modes between studies, differing ventilator strategies and differing methods for measuring and targeting tidal volume. While some of the studies included in the meta-analysis reported a separate subgroup analysis for ELBW or extremely preterm infants (32, 35), others did not. The authors utilized supplementary data from studies to get a total of 247 ELBW babies in the meta-analysis which did not have the power to identify significant differences between the VTV and PLV group (20). Indeed, there were no significant differences in major outcomes noted between VTV and PLV for ELBW babies (Table 1).

**Table 1:** Characteristics and outcome measures of studies with different ventilation strategies in ELBW infants.

**References**	**Design**	**Group**	**Mortality**	**BPD**	**Time on ventilation**	**Hypocarbia**	**Amount of inspired oxygen**	**IVH or PVL**	**Air leak**
**Klingenberg et al**. **(****20****)**(VTV vs. PLV)	Cochrane review (16 parallel and 4 cross-over studies)	All babies	Reduced in VTV group^*^	Reduced in VTV group^*^	Reduced in VTV group^*^	Reduced in VTV group^*^	No significant difference	Reduced incidence of severe IVH in VTV group^*^	Reduced in VTV group^*^
		ELBWsubgroup	No significant difference	No significant difference	No significant difference	No significant difference	No significant difference	No significant difference	No significant difference
**Bhuta and Henderson-Smart (****40****)**(Elective HFJV vs. CV)	Cochrane review (3 RCTs)	All babies	No significant difference	Reduced in HFJV group^*^	Higher in HFJV group	NR	Fewer days on oxygen in HFJV group	Increased risk of PVL in HFJV group	NR
		ELBWsubgroup	NR	NR	NR	NR	NR	NR	NR
**Cools et al**. **(****22****)** (Elective HFOV vs. CV)	Cochrane review (19 RCTs)	All babies	Reduced in HFOV group^*^^a^	Reduced in HFOV group^*^	Meta-analysis not done due to variability	NR	Meta-analysis not done due to variability	Increased risk in 2 trials but not in overall meta-analysis	Increased risk in HFOV group^*^
		ELBWsubgroup	NR	NR	NR	NR	NR	NR	NR
**Craft et al**. **(****21****)** (Elective HFFI vs. CV)	Sy-fi study group (RCT)	ELBW babies	No significant difference	No significant difference	No significant difference	NR	No significant difference	No significant difference	No significant difference
**Rojas-Reyes and Orrego-Rojas (****41****)**(Rescue HFJV vs. CV)	Cochrane review (1 RCT)	All babies	No significant difference	No significant difference	NR	NR	NR	Reduced incidence of new IVH in HFJV group	NR
		ELBWsubgroup	NR	NR	NR	NR	NR	NR	NR

NR, not reported; VTV, volume targeted ventilation; PLV, pressure limited ventilation; HFJV, high frequency jet ventilation; CV, conventional ventilation; HFOV, high frequency oscillatory ventilation; HFFI, high frequency flow interrupter; ELBW, extremely low birth weight – defined as birth weight ≤ 1000gms; Mortality is defined as death before hospital discharge; BPD, bronchopulmonary dysplasia – need for oxygen or ventilatory support at 28 days or 36 weeks postmenstrual age; Hypocarbia defined as CO_2_ tension in arterial blood <40torr; IVH, intraventricular hemorrhage; PVL, periventricular leukomalacia;

* denotes statistically significant difference between two groups (p <0.05);

a *denotes combined death and BPD*.

While we wait for RCTs specifically designed for ELBW infants to be published, it is worth discussing some of the interesting studies related to this topic. Polimeni et al. performed a study with ELBW babies studying the importance of maintaining stable tidal volume from breath to breath (42). The group compared the hypoxemic episodes in VTV vs. PLV group in these babies and showed that while there was no difference in the number of hypoxemic episodes in the two groups, the duration of those hypoxemic episodes was significantly reduced in the VTV group. This was a significant finding as an automatic reaction to these hypoxemic episodes in ELBW babies is to increase the fraction of inspired oxygen (FiO_2_). Frequently these babies would stay on the increased FiO_2_ for some time before the oxygen is titrated down, even though they have recovered from the hypoxemic event. This leads to more oxidative stress for the baby and an increased risk of future lung and eye disease. VTV could partially solve this problem by decreasing the duration of these hypoxemic episodes.

Lista et al. showed that VTV was associated with lower levels of inflammatory cytokines [interleukin 6 (IL−6), interleukin 8 (IL−8), and tumor necrosis factor-alpha (TNFα)] in the tracheal aspirate fluid compared to PLV among preterm infants with respiratory distress syndrome (43). This could be explained by the avoidance of volutrauma and atelectrauma by maintaining a consistent tidal volume over time.

A recently published study by Wong et al. showed that there was a good correlation of measured expired tidal volume through a flow sensor between ELBW babies (<1,000 g) and VLBW babies (1,000–1,500 g) (44). This is a significant finding because VTV relies heavily upon the accurate measurement of expired tidal volume. Indeed, newer ventilators have flow sensors that are very sensitive up to tidal volumes of 5 ml (29). This would encompass a big portion of the tidal volume range seen in ELBW babies weighing <1,000 g. This is supported by the study published by Keszler et al. that a tidal volume of ~5 ml/kg is needed to maintain stable PaCO_2_ in babies weighing <800 g (12).

One concern with VTV for ELBW babies would be the dead space in the flow sensor. While this does not affect ventilation much in larger babies it could be a concern for babies <1,000 g. A study published in 2009 showed that adequate alveolar ventilation was achieved using VTV in babies <800 g in spite of the flow sensor dead space (45). Interestingly, another study with VTV which measured arterial CO_2_ in newborn infants showed that a higher amount of tidal volume was needed to maintain normocapnia in the smallest babies (<500 g) (32). This finding would mean that the extra dead space in the flow sensor may indeed play some role in the smallest micro-preemie population. However, this small disadvantage should not preclude the use of flow sensors in the smallest babies given the multiple advantages it confers, such as measuring accurate tidal volume and flow triggering (46).

## High Frequency Ventilation In Elbw Infants

The goal of high frequency ventilation is to maintain optimal lung expansion while reducing the risk of lung injury by avoiding high or rapid changes in tidal volume (47). Several animal studies have been published over the years demonstrating the advantages of high frequency ventilation over conventional ventilation in immature and/or injured lungs (48, 49). McCulloch et al. showed that maintaining a sustained alveolar expansion through high frequency ventilation prevented lung injury in the atelectasis-prone immature lungs (50). Three forms of high frequency ventilation have been widely used for respiratory management of preterm infants: high frequency oscillatory ventilation (HFOV), high frequency flow interrupter (HFFI) and high frequency jet ventilation (HFJV). Of these three HFOV and HFJV are the two popular modes when compared to HFFI. There are differences in the mechanism between the three modes but the target is the same, that is, to produce optimal gas exchange while minimizing peak and mean airway pressures (51). In HFOV, a piston-like mechanism is used to generate small tidal volumes at the rate of 5–15 Hz (300–900 cycles per minute) which are then transmitted through a rigid circuit, ETT and finally through the tracheobronchial tree to reach the alveoli resulting in gas exchange. The user sets the mean airway pressure (MAP), amplitude and the amount of inspired oxygen based on the need of the patient. In HFOV both inspiration and expiration are active processes (52). HFJV, which is commonly used as a parallel connection with a conventional ventilator, functions differently. The jet ventilator is attached to the ETT through a special 3-way cannula. It generates gas pulses at a high frequency (4–11 Hz or 240–660 cycles per minute) and propels it through one of the ports of the three-way cannula while the conventional ventilator maintains an optimum lung expansion with a stable PEEP (53). HFFI is a recent advancement in some conventional ventilators which offer both conventional and high frequency modes (18). They function similar to the HFJV. A high-pressure system delivers gas into the ventilation-endotracheal tube circuit and a valve system is used to interrupt the flow and produce high frequency breaths. The valve system is controlled either mechanically or through a microprocessor in the ventilator. The operator selects the parameters including the frequency (6–20 Hz or 360–1,200 cycles per minute), PIP and PEEP. While inspiration is an active process in HFJV and HFFI, expiration is passive secondary to lung recoil. Boros et al. performed a study in cats comparing HFOV and HFJV to look at differences in peak and mean airway pressures for similar pH and PaCO_2_ levels (54). They found that HFJV was able to produce better gas exchange compared to HFOV at lower peak and mean airway pressures. This benefit may partly be due to the passive expiration seen in HFJV. While none of the clinical studies have proved one to be better than the other, HFJV is theoretically considered better for non-homogenous lung pathologies and air leak syndromes (55). Both HFOV and HFJV have been used either electively (i.e., as the primary mode of respiratory treatment shortly after birth) or as a rescue (i.e., after the failure of conventional ventilation or a complication from conventional ventilation) in preterm infants (40, 41, 56). It is important to note that Ethawi et al. attempted to perform a Cochrane review comparing HFJV and HFOV for acute pulmonary dysfunction in preterm infants (57). However, they did not find any randomized controlled trials (RCT) or quasi-RCTs that met the inclusion criteria.

## Conventional Ventilation Vs. High Frequency Ventilation In Elbw Infants

There has been much debate regarding the use of conventional ventilation or high frequency ventilation in ELBW babies. Unfortunately, there is not much evidence to suggest that one is superior to the other. As with many other aspects of neonatology, significant practice variability exists among different centers. Bhuta and Henderson-Smart (40) performed a systematic review in 1998 comparing elective HFJV (i.e., starting soon after initiation of mechanical ventilation or shortly after birth) and conventional ventilation in babies <2,000 g or <34 weeks with RDS. While the group did not specifically target the ELBW babies, it is still worth discussing the results. Overall, three trials were included in the review. Of these, one of the trials used both a high (defined as increasing the PEEP by ≥1 cm H_2_O from pre-HFJV baseline and/or using PEEP ≥7 cm H_2_O) and low airway pressure strategy while using HFJV (58) and the other two trials used a low airway pressure strategy (59, 60). The primary outcome was a decreased rate of chronic lung disease without serious adverse effects. The meta-analysis found a reduction in the rate of BPD at 36 weeks corrected age in the HFJV group [Relative risk (RR) 0.58, 95% CI 0.34–0.98]. There were no significant differences in mortality, the overall incidence of IVH or severe IVH (grades 3 and 4), air leaks although the number of ventilator days saw a non-significant increase in the HFJV group (Table 1). There was a non-significant decrease in the number of days on oxygen in favor of the HFJV group. There was no reported data on PDA or hypocarbia. However, one of the trials (60) which used the low airway pressure strategy showed a significantly increased risk for periventricular leukomalacia (PVL) in the HFJV group (RR 5.0, 95% CI 1.19–21.04). The trial that used high mean airway pressure strategy did not show an increased risk of acute brain injuries (58). The authors concluded that HFJV might have an advantage as a primary mode over conventional ventilation in preterm infants but given the adverse effects of HFJV being unclear, more research is needed before clear recommendations can be made.

In 2016, a Cochrane review comparing elective HFOV and CV in preterm infants was published (22). Overall, 19 RCTs between 1989 and 2014 were included. The HFOV group showed a small reduction in the risk of BPD (RR 0.86, 95% CI 0.78–0.96) and combined death or BPD (RR 0.90, 95% CI 0.84–0.97) at 36 weeks corrected age compared to CV. However, the outcomes were variable across different studies. Additionally, the HFOV group had an increased risk of air leak (RR 1.19, 95% CI 1.05–1.34), which may balance out the slight advantage of HFOV in reducing the rate of BPD. Even though two of the included trials reported significantly increased risk of severe IVH in the HFOV group (61, 62), the overall meta-analysis did not find a significant difference between the two groups. There was no statistically significant difference in mortality, the overall incidence of IVH or PVL. Meta-analysis was not done for total ventilator days, duration of oxygen therapy because of high variability between studies (Table 1). Data regarding PDA and hypocarbia were not reported. The sy-fi trial was one of the RCTs included in the review (21). This trial used HFFI as the high frequency mode of ventilation. Interestingly, the sy-fi study was the only RCT with babies <1,000 g. For the purpose of this review, we have discussed this study separately below.

There have been a number of studies over the years comparing the effects of these two modes of ventilation on lung inflammation. Generally, HFV is considered to be less traumatic to the preterm developing lungs when compared to CV. However, the evidence is contradictory. Thome et al. compared levels of numerous inflammatory markers (IL-8, leukotriene B4) in the tracheal aspirates of babies either ventilated with CV and HFOV (63). There was no significant difference in the levels of the inflammatory markers at 10 days of life between the two groups. Lista et al. did a study in 2008 comparing the effect primary VTV and primary HFOV would have on lung inflammation in infants between 25 and 32 weeks gestational age (64). The levels of inflammatory markers (IL-6, IL-8, and TNFα) were measured in the tracheal aspirate on the first, third, and seventh day of life. IL-6 levels were significantly higher in the HFOV group after 3 days. The HFOV group also was found to have longer oxygen dependence. In a similar study published in 2011, the investigators measured the serum levels of Clara cell 16 kD protein (CC16) and IL-6 in babies <30 weeks of gestation age ventilated with either CV or HFOV (65). CC16 and IL6 are considered as biomarkers for alveolar inflammation and leakage. The levels were comparable between the two groups at the third and fourteenth day of life, and at 36 weeks post-menstrual age.

A meta-analysis performed in 2015 by Rojas-Reyes and Orrego-Rojas compared conventional ventilation and rescue HFJV (i.e., after the failure of conventional ventilation mostly after 24 h of life) in babies <35 weeks who had severe pulmonary dysfunction (41). Only one study by Keszler et al. (66) met the inclusion criteria. This study was a multi-center RCT performed between 1987 and 1989 comparing HFJV with conventional ventilation in 144 babies weighing ≥750 g who had developed pulmonary interstitial emphysema (PIE) with the primary outcomes being an improvement of PIE. There was no statistically significant difference in chronic lung disease (RR 0.77, 95% CI 0.54–1.07) or overall mortality (RR 1.03, 95% CI 0.64–1.66) between the two groups. There was no difference between groups regarding air leaks or severe IVH (Table 1). There was a trend toward the decreased incidence of “new” IVH in the HFJV group but it was not statistically significant (RR 0.49, 95% CI 0.19–1.24). Data regarding total ventilator days, PDA, PVL or hypocarbia were not reported and there was no subgroup analysis for babies <1,000 g. Overall, the level of evidence from this study was assessed to be low in quality as the study was performed before the era of surfactant and antenatal steroids making interpretation of the results difficult.

The sy-fi study group performed a RCT between 1999 and 2000 comparing HFFI and conventional ventilation in ELBW babies (21). Forty-six infants were enrolled in the study from two separate centers. There was no significant difference in the incidence of BPD or oxygen requirement at 36 weeks post-menstrual age. The total number of days on a ventilator was similar between the two groups. The study did not find any difference in mortality, duration of oxygen therapy, air leak, severe IVH or PDA between the HFFI and CV in ELBW babies (Table 1). Data on hypocarbia was not reported. A previous study was published by Thome et al. comparing elective HFFI and CV (within 6 h of birth) in preterm infants (67). The study, however, was not specifically done on ELBW babies. There was no significant difference between the two groups for BPD at 30 days (88 vs. 88%) or at 36 weeks post-menstrual age (25 vs. 23%). An even earlier study published in 1993 also used the HFFI in babies <1,800 g with RDS (68). While there was a trend of decreased BPD in the HFFI group compared to the CV group (63 vs. 80% at 28 days, 25 vs. 40% at 36 weeks), the result was not statistically significant.

A meta-regression analysis published by Bollen et al. in 2007 analyzed 15 RCTs performed over the years comparing HFV and conventional ventilation in infants with RDS (69). They found comparable pulmonary outcomes in both groups when they adjusted for the integration of lung-protective ventilation strategies (i.e., avoiding overdistension or atelectasis, surfactant administration, controlled oxygen use) during conventional ventilation over the years and the types of ventilator used. There is a general belief that prolonged time spent on CV before switching to HFV diminishes the benefits of HFV. However, the results of this study were not compatible with that hypothesis. Consequently, at present, making a clear recommendation between HFV and CV especially for ELBW babies is difficult and clinical judgment should be used while deciding between one or the other.

## Conclusion

In conclusion, there is a lot of variations in practice that exist when it comes to the ventilator management of extremely preterm infants. This is due to evolving technology in ventilator modes, performance and its use supporting ELBW infants. At present, no single ventilator has been shown to be superior to others. There is no clear consensus as to which ventilator mode is preferable as the primary mode in ELBW babies with respiratory failure. The several studies discussed in this review illustrate the difficulty in making any clear recommendations. While VTV and HFJV do show some promise in some studies, these studies were not powered to determine significant reductions in mortality or morbidity in ELBW babies. Therefore, larger studies comparing the outcomes of different modes of ventilation as primary support are needed specifically targeting ELBW population.

## Author Contributions

AG and AM wrote the first draft of the manuscript. KS edited and revised the manuscript. All authors approved the final manuscript for submission.

### Conflict of Interest

The authors declare that the research was conducted in the absence of any commercial or financial relationships that could be construed as a potential conflict of interest.
